# Intestinal Transport Characteristics and Metabolism of *C*-Glucosyl Dihydrochalcone, Aspalathin

**DOI:** 10.3390/molecules22040554

**Published:** 2017-03-30

**Authors:** Sandra Bowles, Elizabeth Joubert, Dalene de Beer, Johan Louw, Christel Brunschwig, Mathew Njoroge, Nina Lawrence, Lubbe Wiesner, Kelly Chibale, Christo Muller

**Affiliations:** 1 Biomedical Research and Innovation Platform, South African Medical Research Council, Tygerberg, Cape Town 7130, South Africa; johan.louw@mrc.ac.za (J.L.); christo.muller@mrc.ac.za (C.M.); 2 Plant Bioactives Group, Post-Harvest and Wine Technology Division, Agricultural Research Council, Infruitec-Nietvoorbij, Stellenbosch 7600, South Africa; JoubertL@arc.agric.za (E.J.); DBeerD@arc.agric.za (D.d.B.); 3 Department of Food Science, Stellenbosch University, Stellenbosch 7600, South Africa; 4 Department of Biochemistry and Microbiology, University of Zululand, Kwa-Dlangezwa 3886, South Africa; 5 Department of Chemistry, University of Cape Town, Rondebosch 7701, South Africa; CA.brunschwig@uct.ac.za (C.B.); Mathew.Njoroge@uct.ac.za (M.N.); kelly.chibale@uct.ac.za (K.C.); 6 Drug Discovery and Development Centre (H3D), University of Cape Town, Rondebosch 7701, South Africa; nina.lawrence@uct.ac.za; 7 Division of Clinical Pharmacology, University of Cape Town, Observatory, Cape Town 7925, South Africa; lubbe.wiesner@uct.ac.za; 8 Institute of Infectious Disease and Molecular Medicine, Faculty of Health Sciences, University of Cape Town, Observatory, Cape Town 7925, South Africa; 9 South African Medical Research Council Drug, Discovery and Development Research Unit, University of Cape Town, Rondebosch 7701, South Africa; 10 Department of Medical Physiology, Stellenbosch University, Tygerberg 7507, South Africa

**Keywords:** aspalathin, bioavailability, Caco-2, transport, metabolism

## Abstract

Insight into the mechanisms of intestinal transport and metabolism of aspalathin will provide important information for dose optimisation, in particular for studies using mouse models. Aspalathin transportation across the intestinal barrier (Caco-2 monolayer) tested at 1–150 µM had an apparent rate of permeability (P_app_) typical of poorly absorbed compounds (1.73 × 10^−6^ cm/s). Major glucose transporters, sodium glucose linked transporter 1 (SGLT1) and glucose transporter 2 (GLUT2), and efflux protein (P-glycoprotein, PgP) (1.84 × 10^−6^ cm/s; efflux ratio: 1.1) were excluded as primary transporters, since the P_app_ of aspalathin was not affected by the presence of specific inhibitors. The P_app_ of aspalathin was also not affected by constituents of aspalathin-enriched rooibos extracts, but was affected by high glucose concentration (20.5 mM), which decreased the P_app_ value to 2.9 × 10^−7^ cm/s. Aspalathin metabolites (sulphated, glucuronidated and methylated) were found in mouse urine, but not in blood, following an oral dose of 50 mg/kg body weight of the pure compound. Sulphates were the predominant metabolites. These findings suggest that aspalathin is absorbed and metabolised in mice to mostly sulphate conjugates detected in urine. Mechanistically, we showed that aspalathin is not actively transported by the glucose transporters, but presumably passes the monolayer paracellularly.

## 1. Introduction

Aspalathin, a *C*-glucosyl dihydrochalcone, unique to rooibos (*Aspalathus linearis*) herbal tea, is apart from its antioxidant activity [1], increasingly of interest due to its anti-diabetic effects [2]. Studies in humans showed that the compound is absorbed after drinking rooibos tea [3,4,5]. Eight aspalathin metabolites found in human urine were structurally linked to aspalathin. The presence of aspalathin conjugates formed through glucuronidation, methylation, and sulphation indicated that phase 2 metabolism mainly occurs. Traces of unconjugated aspalathin was found in the plasma of subjects who consumed 500 mL of green rooibos infusion [5].

The hydrophilic nature of aspalathin suggests that it is likely to be transported using active transporters such as the glucose transporters (SGLT1 and/or GLUT2) [6] since the attached carbon–carbon (C–C)-linked glucose is not removed by brush border enzymes [7]. These glucose transporters are of special interest because the efficacy of aspalathin as an anti-diabetic compound might be linked to the inhibition of SGLT2 receptors in the kidneys [8]. Structurally similar compounds like phloridzin and other analogues like the *C*-glucosides canagliflozin or dapagliflozin used as type 2 anti-diabetics are specific SGLT inhibitors (selective inhibition of reabsorption of glucose from urine by SGLT2 in the kidney; and selective inhibition of glucose absorption by SGLT1 in the intestine) [9]. The intestinal transport mechanism of aspalathin has not yet been investigated intensively. Courts and Williamson [10] suggested that passive diffusion of aspalathin occurs across the intestinal epithelial monolayer (Caco-2) without deglycosylation. However, data to support this suggestion were not presented. Furthermore, the bioavailability of aspalathin has only been investigated in the presence of other rooibos constituents as in an extract or infusion, but not as a pure compound. Breiter et al. [5] attributed a disparity in the absorption of aspalathin, when subjects consumed green rooibos infusion and an aspalathin-enriched fraction of green rooibos, reconstituted in water, to matrix and/or synergistic effects.

**Figure 1:**
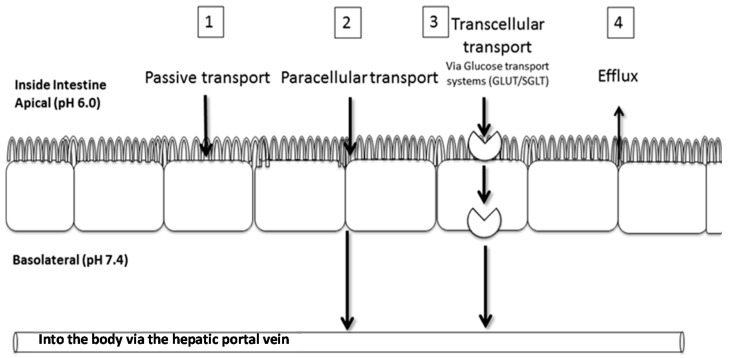
Possible transport pathways of aspalathin across the intestinal epithelium, namely (**1**) passive transcellular; (**2**) paracellular; (**3**) active carrier-mediated and transcytosis; as well as (**4**) carrier-mediated efflux (Adapted from http://www.sddu.leeds.ac.uk/gts/postgrad/resources/poster/Courts.pdf, detailed in [3]).

The Caco-2 cell culture model system, an excellent model for determining the absorption potential of drug candidates, mechanisms of absorption (Figure 1), and excretion of drugs and their metabolites, was used to study the transport of aspalathin, as well as the effect of green rooibos extract and high glucose concentration on aspalathin absorption. In addition, the Caco-2 model was used to elucidate the possible role of SGLT2, GLUT2, and the efflux transport systems (PgP) on the absorption and thus bioavailability of aspalathin. Futhermore, investigation of the metabolism of pure aspalathin in mice will provide useful insights in understanding efficacy data. A mouse model was preferable to rat models due to the smaller quantity of pure compound required. Previous studies by our group on the anti-diabetic effects of aspalathin-enriched green rooibos extract were performed in rat models [2], while the hypoglycaemic effect of aspalathin—isolated from green rooibos—has been studied in type 2 diabetic *db*/*db* and *ob*/*ob* mice models [11,12]. The results obtained in the present study will contribute to an understanding of aspalathin bioavailability relevant to its bioactivity. The knowledge gained should open possibilities for additional chemical manipulation of aspalathin to enhance/improve its efficacy as a therapeutic product.

## 2. Results

### 2.1. Physicochemical Characteristics of Aspalathin

Aspalathin demonstrated high solubility in 0.01 M HCl (pH 2), phosphate buffered saline (pH 6.5) and Fasted State Simulated Intestinal Fluid (FaSSIF) (pH 6.5), while the partition coefficient (log D at pH 7.4) of 0.13, indicated low lipophilicity (Table 1).

### 2.2. Transport of Aspalathin across a Caco-2 Monolayer

Preliminary results showed higher stability for aspalathin in transport buffer (as described in Section 4.3) at pH 6.0 than pH 7.4 (data not shown). In addition, the viability of the Caco-2 cells using pH 6.0 on the apical side and pH 7.4 on the basolateral side, and the integrity of the monolayer, were not significantly affected (data not shown). These conditions mimic the physiological pH of mainly upper sections of the small intestine [13] and the circulatory system, respectively. The maximum concentration of aspalathin in Hank’s balanced salt solution (HBSS) buffer (pH 6.0; apical side) not cytotoxic to Caco-2 cells (>80% viability) after 2 h exposure was 150 µM (Figure S1; supplementary material).

Aspalathin passed through the Caco-2 monolayer intact and non-metabolised as no aspalathin metabolites could be detected by high performance liquid chromatography with diode-array (HPLC-DAD) (Figure S2; supplementary material) or mass spectrometry detection (MS) in the basolateral or apical samples taken after 2 h exposure at 150 µM aspalathin. Low uptake (~5% after 2 h) and a low transport rate (P_app_ = 1.73 × 10^−6^ cm/s) were demonstrated for aspalathin (Table 2). Caffeine, the control, showed high uptake (79.5% after 2 h) and a high transport rate (P_app_ = 6.5 × 10^−5^ cm/s).

Transport of aspalathin across the Caco-2 monolayer in the presence of a high glucose concentration (20.5 mM) was significantly (*p* < 0.05) inhibited (Table 2), suggesting competition between aspalathin and glucose for transport across the monolayer. The presence of the glucose transporters, SGLT1 and GLUT2, as well as the multidrug resistance protein (MDR-1) in the Caco-2 model were confirmed by Western blot analysis (Figure S3; supplementary material). Specific inhibitors were added to establish whether glucose transporters were in fact the primary method of transport of aspalathin. However, phloridzin and phloretin, inhibitors of SGLT1 and GLUT2, respectively, showed no noticeable effect on aspalathin transport (Table 3). In a bi-directional Caco-2 permeability assay, using verapamil hydrochloride, a known multidrug resistance reversal agent and PgP efflux pump inhibitor, an efflux ratio of 1.1 (Table 3) was obtained, indicating no noticeable efflux for aspalathin. Absorption of aspalathin was furthermore not different when present as pure compound or in an aspalathin-enriched green rooibos extract (SB1) or semi-purified fraction (PEF1) (Table 4). The P_app_ values for aspalathin were not significantly different (*p* > 0.1) when compared with the transport of 1 µM and 150 µM aspalathin (Table 4). Irrespective of the matrix, its P_app_ values were directly comparable, as were those of nothofagin, isoorientin, and orientin (Table 4).

### 2.3. Aspalathin Metabolites in Mouse Urine

The fragmentation pattern of aspalathin in negative mode was characteristic of a *C*-glucoside with intense [M – H − 90]^−^ and [M – H – 120]^−^ ions (Table 5). Further fragmentation between *α*- and β-carbons of the dihydrochalcone yielded ions at *m*/*z* 209 and 179. Ions at *m*/*z* 289 and 167 were formed from the cleavage of the C–C bond of the *C*-glucosyl moiety as found previously [14] (Figure S4; supplementary material).

**Figure 2:**
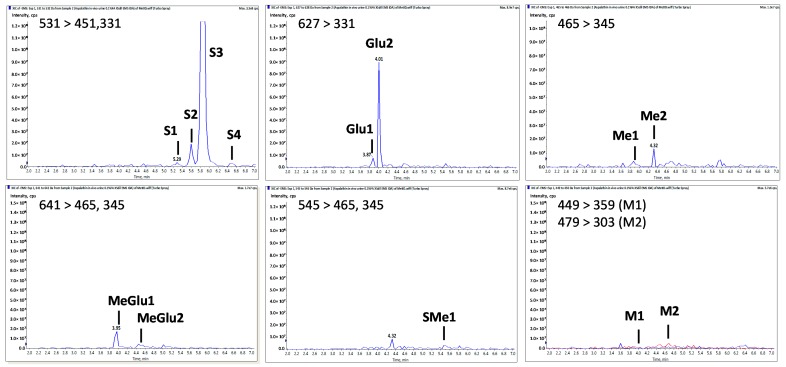
Extracted ion chromatograms of aspalathin metabolites in mouse urine (see Table 5 for peak numbers).

No aspalathin, nor any of its metabolites, could be detected in the blood, while 13 aspalathin metabolites were detected in the urine (Table 5, Figure 2). The metabolites present in the urine were mainly aspalathin sulphates (S1, S2, S3, S4) and aspalathin glucuronides (Glu1, Glu2) in terms of relative concentrations, while methylated metabolites (Me1, Me2) and metabolites derived from a combination of methylation and glucuronidation (MeGlu1, MeGlu2) or methylation and sulphation (SMe1) were also present. S1, S2, S3, and S4 had a [M − H]^−^ at *m*/*z* 531 and characteristic MS^2^ fragments at *m*/*z* 451, 361 and 331, corresponding respectively to the loss of a sulphate moiety (−80 Da) and further fragmentation of the *C*-glucosyl moiety of the parent compound (−90 and −120 Da). Glu1 and Glu2 were identified by their [M − H]^−^ at *m*/*z* 627 and the characteristic fragmentation of the *C*-glucosyl moiety.

Methylated metabolites were identified by their [M − H]^−^ at *m*/*z* 465, while the MS^2^ fragments at *m*/*z* 375 and 345 indicated that methylation occurred on the A or B ring. MeGlu1, MeGlu2, and SMe1 were identified similarly based on their [M − H]^−^ and fragments resulting from cleavage of the glucuronide, sulphate, methyl, and/or C-glucosyl moieties. One metabolite of aspalathin aglycone (M2) was identified as a methyl-*O*-tetrahydroxy-dihydrochalcone-*O*-glucuronide with [M − H]^−^ at *m*/*z* 479 and fragment Z_0_^−^ at *m*/*z* 303, indicating methylation on the A or B ring. Finally, a product from oxidative cyclisation of aspalathin (M1) was identified as a *C*-glucopyranosyl eriodictyol. This compound (M1) was also identified when aspalathin was incubated in phosphate buffer at pH 7.4 and in mouse liver microsomes (Figures S5–S7; supplementary material).

## 3. Discussion

Aspalathin has several molecular features that violate rules for good bioavailability [15]. Its low lipophilicity (log D_7.4_ = 0.13) indicates aspalathin to have a low passive transcellular permeability through lipid bilayers [16]. Another structural feature is the presence of a C–C-linked glucose moiety. Typically, adsorption of lipophilic flavonoid aglycones through passive diffusion follows deglycosylation of *O*-glycosides by lactase phloridzin hydrolase (LPH) localised in the brush-border of the small intestine epithelial cells and by cytosolic β-glucosidase [7]. *C*-glycosides, however, can only be metabolised in the colon by bacterial hydrolases [17] before further microbial degradation. Human studies showed that deglycosylation of aspalathin is not a prerequisite for its absorption when ingested as rooibos tea, but these studies confirmed its poor absorption from rooibos and subsequent extensive phase 2 metabolism [3,4,5]. In spite of this, bioactivity, and in particular anti-diabetic effects have been demonstrated for aspalathin and rooibos extracts in type II diabetes rat [18] and mouse [11,12] models, confirming that aspalathin is indeed one of the major bioactive compounds of rooibos, meriting an understanding of the mechanisms of intestinal absorption. Given interspecies differences in phase 2 drug metabolising enzymes [19], the aspalathin metabolites formed in mice are relevant to studies on the anti-diabetic properties of aspalathin.

We have shown the presence of aspalathin metabolites in mouse urine, indicating that aspalathin was absorbed in the mouse gastrointestinal tract at some point with the C–C bond of the *C*-glucosyl moiety being intact as previously found in humans [5] and in pigs [20]. Aspalathin was then metabolised mainly into sulphates in mice, in either the gut, liver or kidneys. In contrast, methylation and glucuronidation appear to be a dominant biotransformation in humans [3,4,5] and pigs [20], when ingesting rooibos tea or its extract. In vitro, methylation of aspalathin occurred both in rat liver and intestinal cytosolic subcellular fractions, while sulphation of aspalathin occurred in rat liver cytosol [21]. Sulphotransferases are mainly expressed in the liver of rodents [22], and have a pronounced extra-hepatic pattern in humans. These species differences in terms of metabolism have to be considered when extrapolating aspalathin efficacy data from mice to humans, especially if aspalathin metabolites play a role in pharmacological activity at the target tissue.

Identification of a *C*-glucopyranosyl eriodictyol (M1) in mouse urine after ingestion of pure aspalathin shows that oxidative cyclisation of aspalathin may occur in mouse. Previously (*S*)-eriodictyol-8-*C*-glucoside and its (*R*)/(*S*)-6-*C*-isomers were detected in the plasma of subjects drinking rooibos infusion [5].

Since aspalathin is a weak acid and has a molecular weight higher than 400 g/mol (Table 2), it could be classified as class 3B in the Extended Clearance Classification System (ECCS) [16]. Renal clearance is expected to be the rate-limiting mechanism of disposition for this class, consistent with the high levels of aspalathin metabolites found in the mouse urine. The low bioavailability observed for aspalathin in blood in the different species might consequently be due to high renal clearance. High plasma protein binding may also play an important role as *C*-glucosyl flavonoids are known to be highly protein bound [10], which reduces passive transcellular or paracellular permeation [23] and hampers detection of metabolites.

The transport process across absorptive epithelia is mediated through one or several processes (Figure 1). Mechanistically, due to its physical and chemical properties, passive transport of aspalathin was excluded. Following inhibition of aspalathin transport in the presence of a high glucose concentration, the role of active transport by glucose transporters was hypothesised as a primary mechanism of transport across the Caco-2 monolayer. However, inhibition studies demonstrated that aspalathin was transported neither by SGLT1, nor GLUT2, thereby excluding active transport as a primary mechanism of transport. In addition, the low bi-directional permeability of aspalathin indicated that aspalathin was not effluxed from the cells back into the apical chamber, suggesting a paracellular diffusion mechanism. On the other hand, aspalathin consistently passed across the intact Caco-2 monolayer as shown by HPLC-DAD and -MS analysis. The physical barrier properties of the monolayer were confirmed by Lucifer yellow, a low permeability paracellular marker molecule [24].

The unresolved issue is whether aspalathin is crossing the intestinal barrier paracellularly and whether the amount of aspalathin leaking through the intestinal barrier is sufficient to elicit positive biological effects. Passive diffusion through spaces between epithelial cells is regulated by intercellular junctional complexes [25], and diffusion of compounds through these complexes is principally determined by interrelated physicochemical characteristics: lipophilicity, polarity (charge, hydrogen bonding, and polar surface area) and molecular mass [26,27] as described by Fick’s first law of diffusion. The rate-limiting factor in small intestinal permeability is the integrity of tight junctions between the small intestinal enterocytes [28], as well as the physical characteristics of the luminal substances. It is widely accepted that tight junctions are dynamic structures and may be flexible barriers of absorption [25]. It is known that a major portion of intestinal glucose absorption occurs through tight junctions and not by saturable transcellular active transport, chiefly by SGLT1 and GLUT2 [29]. A large set of structural and regulatory molecules control the plasticity and permeability of tight junctions [30]. These structural and regulatory molecules are mainly composed of proteins, including occludin [31], claudins [32], JAM-A (junctional adhesion molecule A) [33], and tricellulin [34]. At high intraluminal glucose concentrations, the osmotic gradient leads to increased permeability and distribution changes of the main proteins (claudins and occludin) of the tight junctions in Caco-2 cells, increasing intercellular leakage [29,35]. However, at high glucose concentrations, competition mechanisms between aspalathin and glucose can occur.

In vitro, the median errors of estimation of intestinal leakage using mannitol and lactulose were 5.0% (range 1.2 to 5.0%) and 1.3% (range 0.2 to 1.3%), respectively [36]. This is consistent with the findings from our experiments where the average passage of aspalathin was 5.0% (range 3.1 and 6.5%) at a glucose concentration of 5.5 mM.

In a cross-over study in humans [5] the flavonoid concentration in the plasma was lower when an aspalathin-enriched fraction of green rooibos (reconstituted in water), instead of green rooibos infusion was consumed, despite the comparable flavonoid content. Huang et al. [37] found that transport of aspalathin from a green rooibos extract solution was slightly, but significantly higher than from an equal concentration of pure aspalathin (i.e., 3.49 ± 1.45 × 10^−6^ cm/s versus 2.48 ± 0.03 × 10^−6^ cm/s). It was postulated that other constituents present in the rooibos extract assist in the transport of aspalathin. These findings differ from ours in terms of the rate of transport, concentration effect and the amount of aspalathin transported across the monolayer. This discrepancy can be attributed to the technical differences in the experimental design, particularly related to lower transepithelial electrical resistance (TEER) readings previously used, indicative of higher permeability of the monolayer. In our Caco-2 study, no effect for the presence of other rooibos constituents could be demonstrated, as the P_app_ value for aspalathin both in the pure, synthesised form, as well as present in the green rooibos extract and semi-purified fraction, were directly comparable. The other major flavonoids present in the extract and fraction—isoorientin, orientin, and nothofagin—have similar P_app_ values to aspalathin. Shi et al. [38] attributed the intestinal transport of orientin and isoorientin predominantly to passive diffusion.

Several metabolic diseases, as well as high caloric diets rich in fat and food additives, impede human epithelial barrier function and increase intestinal permeability resulting in entry of foreign immunogenic antigens and activation of the immune cascade [29]. The metabolic syndrome, for example, which is associated with the development of diabetes and obesity, results from both genetic and environmental factors. Induction of low-grade chronic systemic inflammation, typical of metabolic syndrome [39], can also be linked to intestinal hyperpermeability and increased circulation of endotoxins. In such cases, the passage of potentially beneficial phenolic compounds such as aspalathin, which is shown to have anti-inflammatory properties [40] and a positive ameliorative effect on the associated metabolic disturbances, would be increased.

To our knowledge, this is the first time that the metabolites of aspalathin in mice have been identified. Aspalathin absorption and metabolism have been characterised using pure, synthetic aspalathin, as opposed to aspalathin present in green rooibos extract or a semi-purified fraction of the extract. Sulphated, glucuronidated, and methylated derivatives were detected in the urine of mice, but not in their plasma. Insight was also gained into the mechanism of its intestinal transport using the Caco-2 model. In vitro, aspalathin was able to pass through an intact Caco-2 monolayer. However, the percentage passage is small (~5%) and the rate of transport is indicative of a low bioavailability. Active transport by the enterocytic facultative glucose transporters (SGLT1 and GLUT2), as well as efflux mechanisms (PgP), were shown not to play a role in aspalathin transport. The rate of transport and physical characteristics of aspalathin strongly suggest that aspalathin is transported paracellularly.

## 4. Materials and Methods

### 4.1. Reagents and Extracts

All cell culture supplies were from Corning (New York, NY, USA) unless otherwise specified. Caco-2 cells were obtained from the European Collection of Cell Cultures (cat # 86010202; Salisbury, UK). The Millicell-ERS volt ohmmeter was obtained from Millipore Corporation (Boston, MA, USA) and transwell inserts and 6-well plates from SPL Life Sciences (Pocheon-si, Gyeonggi-do, Korea). Verapamil hydrochloride, phloridzin, phloretin and caffeine were purchased from Sigma-Aldrich (St. Louis, MO, USA). Aspalathin (>95% purity, isolated from *Aspalathus linearis*; for HPLC-DAD quantification), from the PROMEC unit of the Medical Research Council of South Africa (Parow, Cape Town, South Africa), isoorientin from Extrasynthese (Genay, France) and orientin from Carl Roth (Karlsruhe, Baden-Württemberg, Germany). Synthetic aspalathin (99% purity; for Caco-2 experiments and mouse study) was produced by High Force Research Ltd. (Durham, England, UK), based on a scalable synthesis [41]. Eagle’s Minimum Essential Medium (EMEM), pyruvate, non-essential amino acids, l-glutamine, trypsin-EDTA, and penicillin/streptomycin were purchased from Lonza (Basel, Switzerland), Hyclone foetal bovine serum (FBS) from Thermo Scientific (Waltham, MA, USA) and FaSSIF-V2 powder from Biorelevant.com (London, England, UK). Acetonitrile for HPLC and LC-MS were obtained from Sigma-Aldrich and Honeywell, Burdick & Jackson (Muskegon, MI, USA), respectively. All other solvents and reagents of analytical grade were obtained from Sigma-Aldrich.

An aspalathin-enriched organic solvent-based green rooibos extract (SB1; ca 18% aspalathin) [18] and a semi-purified fraction (PEF1; ca. 45% aspalathin) [42] were used to test matrix effects on the absorption of aspalathin in Caco-2 cells.

### 4.2. Physicochemical Characterisation

The kinetic solubility assay was performed using a miniaturised shake flask method. A 10 mM stock solution of aspalathin was used to prepare calibration standards (10–220 µM) in dimethyl sulphoxide (DMSO), and to spike (1:50) duplicate aqueous samples of phosphate buffered saline (pH 6.5), 0.01 M HCl (pH 2), and FaSSIF (pH 6.5), with a final DMSO concentration of 2%. After shaking for 2 h at 25 °C, the solutions were filtered (multiscreen filter plates, 0.45 µm low-binding, hydrophilic polytetrafluoroethylene (PTFE), Merck) and analysed by means of HPLC-DAD (Agilent 1200 Rapid Resolution HPLC; Agilent Technologies, Inc., Santa Clara, CA, USA). Best fit calibration curves were constructed using the calibration standards, which were used to determine the solubility of the aqueous samples [43].

For determination of the lipophilicity of aspalathin, a 10 mM stock solution prepared in DMSO was used to spike (100 µM) a 1:1 mixture of phosphate buffer (pH 7.4) and *n*-octanol. The solutions were shaken vigorously (1500 rpm) on an orbital shaker for 4 h at room temperature. Thereafter, the samples were centrifuged (3500 rpm, 2700× *g*) in order to fully separate the two immiscible fluids. The samples analysed using HPLC-DAD (Agilent 1200 Rapid Resolution HPLC) and the partition coefficient, LogD_7.4_ calculated [23,44].

### 4.3. Caco-2 Transport Experiments

Validation of the Caco-2 transport experiments required consideration of three parameters, i.e., firstly, to determine the cytotoxic concentrations of synthetic aspalathin, secondly, the concentration of aspalathin required to ensure HPLC detection in the basolateral compartment, and finally, to assess whether the transport of aspalathin is concentration dependent. Preliminary Caco-2 experiments were carried out to determine the cytotoxic concentration of synthetic aspalathin (Figure S1; Supplementary Materials), as well as the stability of aspalathin (in an aspalathin-enriched extract, SB1) in the transport medium (4-(2-hydroxyethyl)-1-piperazineethanesulfonic acid) (HEPES) buffer at pH 6 and 7.4).

Cells were cultured at 37 °C and 5% CO_2_ in 95% humidified air in Eagle’s Minimum Essential Medium (EMEM) containing sodium 100 mM pyruvate, supplemented with 2 mM L-glutamine, 1% non-essential amino acids and 10% heat inactivated FBS. Cell cultures were split when 70%–80% confluent, using trypsin/ethylenediaminetetraacetic acid (EDTA). The culture medium, pre-warmed to 37 °C, was refreshed every 2–3 days. Cells were seeded at 1 × 10^4^ cells/cm^2^ and a sub-cultivation ratio of 1:3 was routinely used. Cells were seeded with the addition of 1% penicillin/streptomycin mixture (10,000 units/mL potassium penicillin and 10,000 µg/mL streptomycin sulphate) at 4 × 10^4^ cells/cm^2^ onto 0.4 µm polycarbonate 6-well inserts with an insert area of 4.52 cm^2^. Cells were used for transport experiments when TEER values across wells were >300 Ω, typically 21–23 days post confluence. Only passages 50–60 were used to prevent phenotypic drift.

The cell monolayers were equilibrated briefly with transport buffer, pH 6.0 (HBSS with 2-(*N*-morpholino) ethanesulphonic acid (MES)) on the apical side and pH 7.4 (HBSS with HEPES) on the basolateral side, to remove traces of the culture media. Following this, transport buffers were added for 30 min prior to the addition of treatment (inhibitors were added during this step when required, as well as during the subsequent treatment incubation step). After equilibration, the plates containing transport medium were incubated for 30 min at 37 °C at 5% CO_2_ in 95% humidified air and the TEER value of the monolayer determined. Following removal of the transport medium for apical-to-basolateral compartment studies, 1.5 mL treatment—caffeine, 260 µM; aspalathin, 1–150 µM; SB1, 0.375 mg/mL (ca. 150 µM aspalathin); or PEF1, 0.15 mg/mL (ca. 150 µM aspalathin)—in HBSS (pH 6.0) was added to the apical compartment. For basolateral-to-apical compartment studies, 2.4 mL of treatment at the same concentrations in HBSS (pH 7.4), was added to the basolateral compartment. Caffeine has high permeability, ranking as class 2 in the ECCS [16], and its transport is transcellularly mediated by passive diffusion [45].

To assess whether aspalathin was co-transported with glucose, its transport (100 µM) at both high (20.5 mM) and low (5.5 mM) glucose concentrations, mimicking the intestinal lumen glucose concentrations pre- and post-meals [46,47], was monitored. This was followed by experiments performed in the presence and absence of SGLT1 (100 µM phloridzin), GLUT2 (100 µM phloretin) and efflux, PgP (100 µM verapamil) inhibitors. The presence of SGLT1, GLUT2, and PgP (MDR-1) transport proteins in our Caco-2 cell model were confirmed by Western blot (Figure S3; supplementary material).

Samples (1.2 mL) were withdrawn from the basolateral or the apical compartment to measure the concentration of aspalathin uptake and efflux, respectively at 0, 0.5, 1, 1.5, and 2 h under sink conditions (the sample aliquot replaced with equal volume of relevant transport buffer). Sample aliquots (350 µL) were frozen in liquid nitrogen after the addition of ascorbic acid (35 µL of 10% solution) and stored at −65 °C until HPLC analysis. Results were corrected for dilution and expressed as cumulative transport (% of initial dose) as a function of time. P_app_ (cm/s) for bi-directional transport studies was calculated as previously described [48,49]. Transport of other major flavonoids in the matrices, i.e., nothofagin, the 3-deoxy analogue of aspalathin and its flavone derivatives, orientin and isoorientin, were also determined.

Lucifer yellow (final concentration 0.1 mM) was used as a membrane integrity marker and co-incubated with the compounds and extracts. A typical intact, differentiated monolayer, with tight junctions comparable with that of the small intestine in vivo, was assumed when the percentage of Lucifer yellow passing across the membrane was <3% [50].

HPLC-DAD analysis of samples from the apical and basolateral compartments was performed in duplicate on an Agilent 1200 system as previously described [42]. Basolateral and apical samples taken after 2 h with pure aspalathin were also analysed using LC-MS (parameters described in [42]) to determine whether aspalathin metabolites were formed in the Caco-2 model.

All experiments were performed in triplicate and the data expressed as mean ± SD, unless otherwise indicated. Differences between mean values were analysed by means of a Student’s paired two tailed *t*-test and considered significant if *p* < 0.05.

### 4.4. In Vivo and In Vitro Metabolism of Aspalathin

#### 4.4.1. Treatment and Sample Collection

An acute mouse study was performed under ethical approval from the Ethics Committee for Research on Animals (ECRA) of the South African Medical Research Council (Ref:07/13). Three 8-week old male C57.BKS mice, obtained from the Primate Unit of the South African Medical Research Council (Tygerberg, South Africa), were housed individually in polycarbonate cages with wired lids and kept in a controlled environment of 23–24 °C, 50% humidity, and a 12 h light/dark cycle. Prior to the study, urine was collected from the fasting mice overnight in a metabolic cage. Baseline fasted blood was collected via tail snip into a heparin tube, snap frozen in liquid nitrogen, and stored at −80 °C. Each mouse received a single dose of synthetic aspalathin (50 mg/kg body weight) dissolved in phosphate-buffered saline (PBS) (5 mg/mL) via orogastric gavage. Blood samples were collected and handled as described for fasting at time 0 (pre-gavage), and 1, 2, 4, 6, and 8 h post-gavage. All the samples were collected into the same heparin tube and kept on ice during the experiment. Urine was collected over the 8-h post-gavage monitoring period and the samples were stored at −80 °C until analysis.

Synthetic aspalathin (10 µM) was incubated at 37 °C in a solution containing 1 mg/mL microsomes (male Mouse BALB/c, Xenotech, Kansas city, KS, USA), magnesium chloride (5 mM) and nicotinamide adenine dinucleotide phosphate (NADPH) (1 mM) in phosphate buffer (100 mM, pH 7.4), for 1 h while shaking. A control containing all the sample constituents (not incubated), another containing no NADPH, and another containing only the compound in phosphate buffer were also included and handled similarly to the test sample.

#### 4.4.2. Metabolite Identification by MS

The frozen whole blood samples, as well as the samples incubated with microsomes and controls, were thawed, extracted by protein precipitation using 0.1% formic acid (FA) in acetonitrile, centrifuged and filtered through 0.22 µm polyvinylidene difluoride (PVDF) filters (Merck Millipore, Frankfurter Straße, Darmstadt, Germany). Frozen whole urine samples were thawed and diluted 5 times in 0.1% aqueous FA. Samples were analysed by LC-MS/MS on an Agilent 1200 Rapid Resolution HPLC system (600 bar, Agilent Technologies) coupled to a 4000 QTRAP^®^ (AB Sciex, Framingham, MA, USA) equipped with a Turbo V™ ion. Filtered samples (10 μL), stored on a sample tray maintained at 8 °C, were injected on a Gemini C6-Phenyl column (150 × 2.0 mm, 3 µm particles) (Phenomenex, Santa Clara, CA, USA) at 40 °C. Metabolites were separated using gradient separation at 0.4 mL/min with 0.1% aqueous FA (A) and 0.1% FA in acetonitrile (B): 5% B, 0–1 min; 5%–50% B, 1–3 min; 50% B, 3–6 min; 50%–100% B, 6–7 min; 100% B, 7–10 min (equilibration at initial conditions for 5 min).

All mass scans were performed using electrospray ionisation in negative mode, with the following operating parameters: curtain gas, 30 psi; nebuliser gas, 50 psi; turbo gas, 60 psi; source temperature, 400 °C; ion spray voltage, −4.5 kV; declustering potential, −75 V; collision activated dissociation gas setting, high; scan speed, 4000 Da/s. Enhanced mass spectrum was used as a survey scan to trigger information dependent acquisition of MS/MS spectra of the two most intense peaks exhibiting counts higher than 100,000 cps. Collision energy and spread were set at −35 V and 25 V, respectively. A neutral loss scan for 120 Da and a precursor ion scan for *m*/*z* 331 were also performed using the following parameters: entrance potential, −10 V; collision energy, −22 V; collision cell exit potential, −7 V.

Aspalathin metabolites were identified using Lightsight v2.3 (AB Sciex, Vaughan, ON, Canada) by comparison of the chromatograms over time and in the NADPH-free control for in vitro samples (microsomes). For the in vivo samples (mouse blood and urine), chromatograms were compared to the pre-treatment controls. The tentative identity of the metabolite was deduced by comparison of the product ion spectra of the pseudomolecular ion ([M − H]^−^) of the metabolite with that of aspalathin using Analyst 1.6 (AB Sciex).

## Figures and Tables

**Table 1:** Physicochemical properties of aspalathin used to predict bioavailability.

Physicochemical Properties	Values
Molecular weight (g/mol)	452.1
Kinetic solubility (µM) at pH 2, pH 6.5 and in FaSSIF at pH 6.5	153, 123, 119
Log D at pH 7.4	0.13

Abbreviation: FaSSIF Fasted State Simulated Intestinal Fluid.

**Table 2:** Apparent permeability and % passage (calculated as a direct measure of concentration) of aspalathin (150 µM) and caffeine (260 µM) across the Caco-2 monolayer.

Treatment	P_app_ a-b (cm/s) ^c^	P_app_ b-a (cm/s) ^c^	% Passage
Caffeine (*n* = 9) ^a^	6.50 ± 0.99 × 10^−5^	7.23 ± 0.23 × 10^−5^	79.50 ± 1.23
Aspalathin (*n* = 18) ^a^	1.73 ± 0.97 × 10^−6^	2.15 ± 0.23 × 10^—6^; efflux ratio 1.1	4.95 ± 2.11 ^d^
Aspalathin with high glucose (*n* = 8) ^b^	2.90 ± 0.75 × 10^−7^	N/A	2.34 ± 2.35 ^d^

^a^ 5.5 mM glucose; ^b^ 20.5 mM glucose ^c^ Apparent permeability values for apical to basolateral (a-b) and basolateral to apical (b-a) transport studies; ^d^ Values differ significantly (*p* < 0.05).

**Table 3:** Effect of various inhibitors on rate of transport of aspalathin (100 µM).

Treatment	Inhibited Protein	P_app_ a-b ^b^ − Inhibitor	P_app_ a-b ^b^ + Inhibitor	P_app_ b-a ^b^ − Inhibitor	P_app_ b-a ^b^ + Inhibitor	Efflux Ratio − Inhibitor	Efflux Ratio + Inhibitor
Phloridzin	SGLT1	1.73 ± 0.97	1.47 ± 1.10 ^c^	2.09 ± 0.23	1.47 ± 0.9 ^c^	1.21	0.99 ^c^
Phloretin	GLUT2	1.73 ± 0.97	1.67 ± 0.43 ^c^	2.09 ± 0.23	1.95 ± 1.1 ^c^	1.21	1.33 ^c^
Verapamil	PgP	1.73 ± 0.97	1.84 ± 0.20 ^c^	2.09 ± 0.23	1.94 ± 0.68 ^c^	1.21	1.1 ^c^

^a^ 100 µM; ^b^ ×10^−6^ cm/s; ^c^ There was no noticeable effect on the rate of transport in the presence of the inhibitors.

**Table 4:** Rate of transport and concentrations tested of aspalathin from a buffered solution of synthetic aspalathin, as well as aspalathin and other major flavonoids from buffered solutions of aspalathin-enriched green rooibos extract (SB1) and semi-purified fraction (PEF1).

Treatment	Concentration	P_app_ (cm/s) a-b × 10^−6^
Caffeine	260 µM	67.88 ± 0.99
Aspalathin	1 µM	2.28 ± 0.09
	150 µM	1.73 ± 0.97
SB1 Aspalathin	0.38 mg/mL ^a^	2.00 ± 1.10
SB1 Nothafagin		1.92 ± 1.10
SB1 Isoorientin		1.81 ± 1.10
SB1 Orientin		1.99 ± 1.30
PEF1 Aspalathin	0.15 mg/mL ^b^	2.11 ± 0.20
PEF1 Nothofagin		2.22 ± 0.30
PEF1 Isoorientin		1.69 ± 0.20
PEF1 Orientin		1.92 ± 0.20

^a^ SB1 concentration in transport medium, corresponding to 153, 11, 17, and 9 µM aspalathin, nothofagin, isoorientin, and orientin, respectively; ^b^ PEF1 concentration in transport medium, corresponding to 149, 21, 12, and 8 µM aspalathin, nothofagin, isoorientin, and orientin, respectively.

**Table 5:** Tentative identification of aspalathin metabolites in mouse urine after an oral dosage of 50 mg/kg.

Peak No.	*t*_R_^a^	[M − H]^−^ (*m*/*z*)	MS^2^ Fragment ioNs (*m*/*z*)						Tentative Identity
			[M − H − 176]^−^	[M − H − 80]^−^	Sugar Moiety Cleavage (^0,3^X_0_^−^, ^0,2^X_0_^−^)	α-β Cleavage (X_0_^α,β^ A^−^)	Z_0_^−^, Z_0_^α,β^, A^−^	Other Fragments	
Asp	4.20	451			361, 331	209, 179	289, 167	239, 125	Aspalathin ^b^
S1	5.30	531		451	361, 331		289		Aspalathin-*O*-SO_3_H
S2	5.60	531		451	361, 331	209, 179	289, 167	239, 125	Aspalathin-*O*-SO_3_H
S3	5.85	531		451	361, 331	209, 179	289, 167	239, 125	Aspalathin-*O*-SO_3_H
S4	6.50	531			361, 331				Aspalathin-*O*-SO_3_H
Me1	3.90	465			345	209	289	251	Me-*O*-Aspalathin
Me2	4.30	465			375, 345	209, 179	303, 167	447, 125	Me-*O*-Aspalathin
SMe1	5.50	545		465	375, 345		303		Me-*O*-Aspalathin-*O*-SO_3_H
Glu1	3.90	627			507			331	Aspalathin-*O*-Gluc
Glu2	4.00	627						331, 269	Aspalathin-*O*-Gluc
MeGlu1	3.95	641	465		375, 345	209	303, 167	447, 259, 125	Me-*O*-Aspalathin-*O*-Gluc
MeGlu2	4.50	641	465		375, 345	209	303, 167	447, 125	Me-*O*-Aspalathin-*O*-Gluc
M1	4.05	449			359, 329	223, 193			Eriodictyol-*C*-glucoside
M2	4.80	479	303				167		Me-*O*-tetrahydroxy-dihydrochalcone-*O*-Gluc

^a^ retention time (min); ^b^ not detected in mouse urine, data shown for comparative purposes. Abbreviations: Gluc, glucuronide; Me, methyl; SO_3_H, sulphate.

## Supplementary Materials

Supplementary materials are available online.
