# Supplementary material for: Intestinal Transport Characteristics and Metabolism of C-Glucosyl Dihydrochalcone, Aspalathin
Source: Molecules. 2017 Mar 30;22(4):554. doi: 10.3390/molecules22040554 (PMC6154319; doi:10.3390/molecules22040554)
Supplement: Supplementary file 1 [file molecules-22-00554-s001.pdf]

Supplementary material for:

Intestinal Transport Characteristics and Metabolism of *C*-Glucosyl Dihydrochalcone, Aspalathin

Sandra Bowles [1,*], Elizabeth Joubert [2,3], Dalene de Beer [2,3], Johan Louw [1,4], Christel Brunschwig [5,6,7], Mathew Njoroge [5,6,7], Nina Lawrence [6], Lubbe Wiesner [7], Kelly Chibale [5,6,8,9], and Christo Muller [1,4,10]

1 Biomedical Research and Innovation Platform, South African Medical Research Council, Tygerberg, Cape Town, 7130, South Africa; johan.louw@mrc.ac.za (J.L.); christo.muller@mrc.ac.za (C.M.)
2 Plant Bioactives Group, Post-Harvest and Wine Technology Division, Agricultural Research Council, Infruitec-Nietvoorbij, Stellenbosch 7600, South Africa; JoubertL@arc.agric.za (E.J.); DBeerD@arc.agric.za (D.d.B.)
3 Department of Food Science, Stellenbosch University, Stellenbosch 7600, South Africa
4 Department of Biochemistry and Microbiology, University of Zululand, Kwa-Dlangezwa 3886, South Africa
5 Department of Chemistry, University of Cape Town, Rondebosch 7701, South Africa; CA.brunschwig@uct.ac.za (C.B.); Mathew.Njoroge@uct.ac.za (M.N.); kelly.chibale@uct.ac.za (K.C.);
6 Drug Discovery and Development Centre (H3D), University of Cape Town, Rondebosch 7701, South Africa; nina.lawrence@uct.ac.za
7 Division of Clinical Pharmacology, University of Cape Town, Observatory, Cape Town 7925, South Africa; lubbe.wiesner@uct.ac.za
8 Institute of Infectious Disease and Molecular Medicine, Faculty of Health Sciences, University of Cape Town, Observatory 7925, Cape Town, South Africa
9 South African Medical Research Council Drug, Discovery and Development Research Unit, University of Cape Town, Rondebosch 7701, South Africa
10 Department of Medical Physiology, Stellenbosch University, Tygerberg 7507, South Africa
* Correspondence: sandra.bowles@mrc.ac.za; Tel./Fax: +27-219-380-479

1. Caco-2 Transport Experiments

1.1. Cytotoxicity

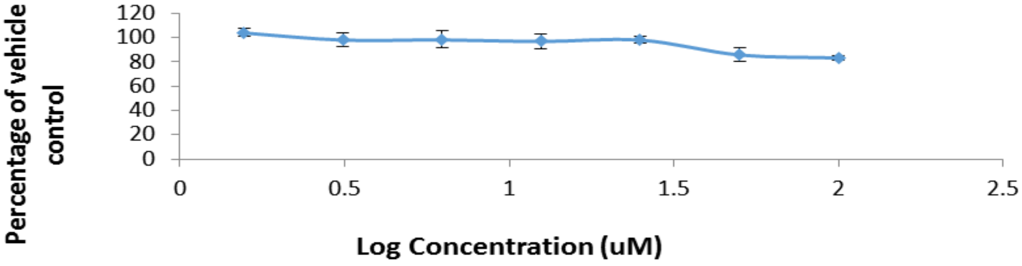

Figure S1: The cytotoxic effects of aspalathin at various concentrations in HBSS buffer (pH 6.0) on Caco-2 cells was assessed by the Vialight™ plus kit. Caco-2 cells were seeded at a density of 4 x 10^4 cells/cm^2 into white clear bottomed 96-well plates and cytotoxicity was assessed according to the manufacturer's recommendation after 12–13 days.

1.2. Analytical Conditions of High-Performance Liquid Chromatography with Diode-Array Detection (HPLC-DAD) with Example Chromatograms.

HPLC-DAD analysis was performed on an Agilent 1200 system (Agilent Technologies, Inc., Santa Clara, CA) equipped with an in-line degasser, quaternary pump, autosampler, column thermostat, and DAD, controlled by Chemstation software (Agilent Technologies, Waldbronn, Germany). The chromatographic conditions were as follows: Separation was performed at 30 °C on a Poroshell SB-C18 column (50 × 4.6 mm, 2.7 µm particle size; Agilent Technologies, Inc.) protected by an Acquity UPLC in-line filter (Waters; 0.2 µm) and an Acquity UPLC VanGuard pre-column (Waters; stationary phase: BEH C18 1.7 µm). The flow rate was 1.0 mL/min, and a multilinear gradient was performed as follows: 0.0–10.0 min, 12.4–16.6%B; 10.0–10.5 min, 16.6–80.0% B; 10.5–11.5 min, 80.0% B; 11.5–12.0 min, 80.0–12.4% B; 12.0–16.0 min, 12.4% B, with solvents A and B being acetonitrile and 0.1% aqueous formic acid, respectively. UV spectra were recorded between 220 and 450 nm.

Stock solutions of the phenolic standards were prepared in dimethylsulfoxide (DMSO) at concentrations of approximately 1 mg/mL and diluted with water according to experimental requirements. All diluted solutions were filtered through 0.22 µm polyvinylidene difluoride (PVDF) filters (Merck Millipore) prior to use.

Six-point calibration curves were set up for all standards. The calibration mixtures were injected at different injection volumes, giving on-column levels of 0.008–1.7 µg for aspalathin, 0.04–0.8 µg for caffeine, and 0.01–0.2 µg for both isoorientin and orientin. Nothofagin was quantified using a previously determined response factor applied to the aspalathin calibration curve. Linear regression, using the least-squares method (Microsoft Excel 2013, Microsoft Corporation, Redmond, WA), was performed on the calibration curve data for each compound to determine the slope and y-intercept. Dihydrochalcones (aspalathin and nothofagin) and caffeine were quantified using their respective peak areas at 288 nm, while the flavones (isoorientin and orientin) were quantified at 350 nm.

(a)

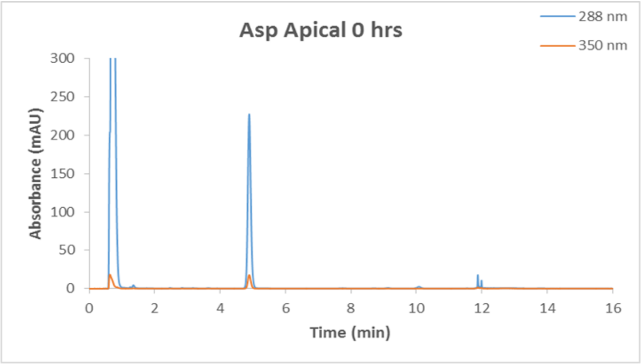

(b)

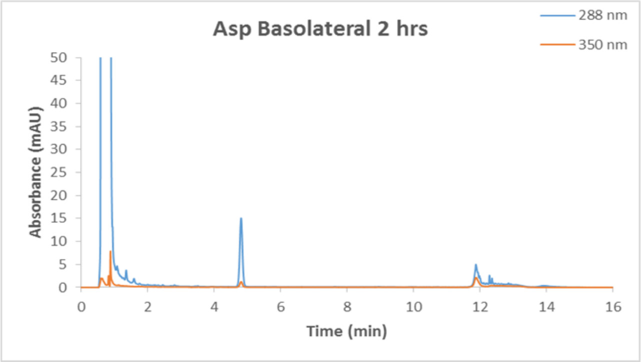

Figure S2: Example HPLC-DAD chromatograms of (**a**) apical (time = 0 h) and (**b**) basolateral (time = 2 h) samples taken from Caco-2 monolayers after incubation with aspalathin.

1.3. Protein Detection

Protein was isolated from differentiated Caco-2 monolayer cell lysates. The total protein content was determined and analysed by SDS-PAGE. 50 ug of total protein was denatured and loaded onto a 10% SDS-polyacrylamide gel and transferred to a PVDF membrane, using a wet-blot system. Non-specific binding was blocked, using 5% w/v low-fat milk in Tris-buffered saline with Tween-20 (10 mmol/L Tris-HCl, pH 7.5, 200 mmol/L NaCl, 0.05% Tween-20) at room temperature for 2 h. Subsequently, the membrane was incubated overnight at 4°C in the presence of the following primary antibodies: anti- SGLT1 ab14686 (1:1000 dilution), anti-GLUT2 ab54460 (1:1000), anti-MDR sc55510 (1:1000), and carnitine palmitoyltransferase (CPT1; 1:1000) with the relevant horseradish peroxidase conjugated secondary antibodies applied the following day for 90 min at room temperature. β-Actin (1:4000) antibody was added as a loading control. Proteins were detected and quantified using a Chemidoc-XRS imager and Quantity One software (Bio-Rad Laboratories, Hercules, CA, USA).

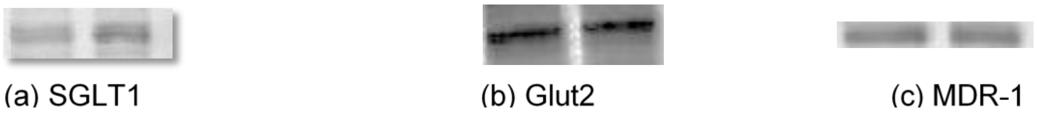

Figure S3: Western Blot analysis of (**a**) SGLT1 (72 kDa), (**b**) GLUT2 (60 kDa), and (**c**) MDR-1 (170 kDa).

1.4. *Stability of Aspalathin.*

In order to determine the stability of aspalathin in the buffer employed for the Caco-2 assay, the aspalathin-enriched extract (SB1) were incubated in sealed vials with deionised water, HEPES buffer pH 6 and HEPES buffer pH 7.4, respectively. The aspalathin content were determined for these samples prior to incubation and after 2 h incubation. The percentage aspalathin degradation obtained in deionised water, HEPES buffer at pH 6 and HEPES buffer at pH 7.4 were 1, 10, and 11%, respectively.

2. In Vivo Metabolism of Aspalathin

2.1. *Fragmentation of Aspalathin in Negative Mode*

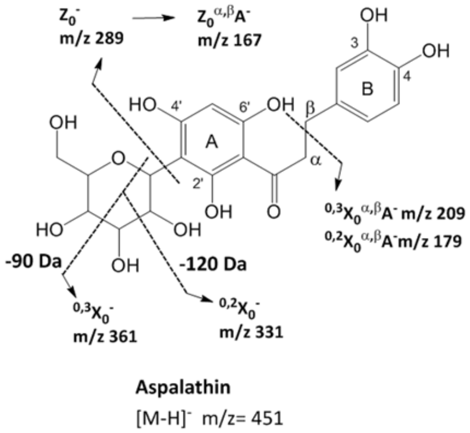

Figure S4: Diagnostic ions for the negative product ion spectra fragmentation of aspalathin and its metabolites

2.2. *In Vitro Incubation of Aspalathin in Mouse Liver Microsomes*

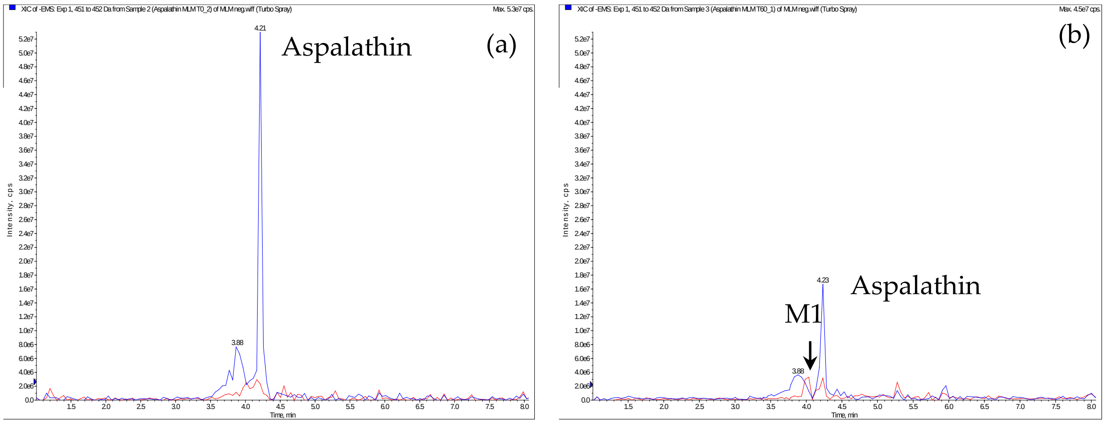

Figure S5: Extracted ion chromatograms of aspalathin and its products formed in (a) no incubation control and (b) mouse liver microsome incubation.

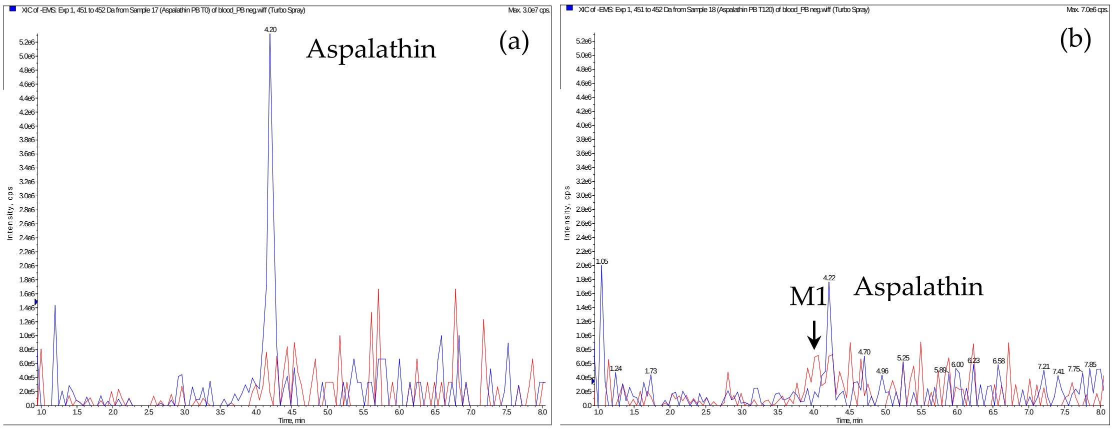

Figure S6: Extracted ion chromatograms of aspalathin and its products formed in the (a) no incubation control and (b) phosphate buffer incubation.

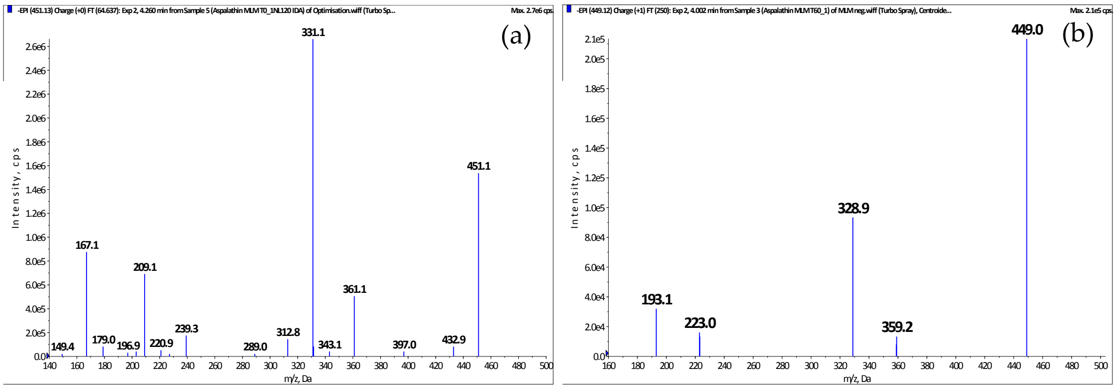

Figure S7. Product ion spectrum of the [M–H]⁻ ion of (a) aspalathin and (b) M1 (identified as *C*-glycosyl eriodictyol).
